# Atypical clinical presentation of systemic juvenile idiopathic arthritis or Still's disease: a report of two cases

**DOI:** 10.11604/pamj.2020.36.12.21932

**Published:** 2020-05-12

**Authors:** Emeline Tiogouo, Paul Eloundou, Hermine Moukodi, Leo Fozeu, Guy Sadeu Wafeu

**Affiliations:** 1 Pediatric Unit, Efoulan District Hospital, Yaounde, Cameroon; 2 Department of Internal Medicine and Specialties, Faculty of Medicine and Biomedical Sciences, Yaounde, Cameroon; 3 Centre for Research on Filariasis and Other Tropical Diseases, Yaounde, Cameroon

**Keywords:** Child, arthritis, Still’s disease, fever

## Abstract

Juvenile idiopathic arthritis (JIA) constitutes a group of arthritis of unknown origin that begins before the age of 16 years. Still´s disease is the systemic form of this condition. Its clinical presentation is marked by fever, rash and sometimes joint pain, in the absence of evidence of another aetiology of the fever. We present the cases of two boys aged 4 and 10 years admitted for fever, with a cerebral origin for the first and no infectious site for the second. Fever persisted after antimalarial treatment and adequate antibiotics. Ferritinaemia, elevated sedimentation rate, lactate dehydrogenase (LDH), triglycerides, and increased serum transaminases, all in the absence of evidence of other inflammatory or malignant diseases were suggestive of Still's disease. Both children received a corticosteroid therapy with progressive dose reduction associated to methotrexate during treatment. Fever disappeared within a few hours after initiation of corticosteroid therapy, with considerable improvement in clinical state. To the best of our knowledge, these cases are among the rare cases of childhood Still disease reported in sub-Saharan Africa. These cases highlight the importance of investigating non-infectious causes of persistent fever in children, in a context of infectious disease endemicity.

## Introduction

Juvenile idiopathic arthritis (JIA) is the most common chronic rheumatic disease in children with about 1 in 1000 children affected worldwide. There are 07 groups of this pathology which have been described. The frequency of JIA varies from one geographic region to another. There is a predominance of oligoarthritis in Western countries, systemic arthritis in Asia, polyarthritis in India, Costa Rica and South Africa. [1]. Still disease is the systemic form of JIA and its causes remain unknown. Given the polymorphic nature of pathologies constituting JIA and their complex pathogenesis, they are distinguished by their clinical and biological presentation.

Systemic juvenile idiopathic arthritis (SJIA) is distinguished by a systemic involvement and a particularly severe inflammatory syndrome. Its diagnosis is based on major criteria such as a hectic fever, a typical rash during febrile peaks, both associated or not with symmetrical or non-symmetrical polyarticular involvement. Minor criteria such as dysphagia, odynophagia, poly lymphadenopathy, hepatomegaly and splenomegaly may complete the picture [2]. On paraclinical, it is common to note an increase in ferritinaemia with a fall in its glycosylated beta fraction, very high C-reactive protein (CRP) and sedimentation rate indicating significant biological inflammation. In addition, hepatic cytolysis, increased lactate dehydrogenase (LDH) and hypertriglyceridemia are not uncommon.

The diagnosis is made after excluding other possible causes of fever in children [2, 3]. This is because fever is the major symptom of several diseases such as malaria and various bacterial infections; infections which are very frequent in children in low/middle income countries like Cameroon. This situation leads to excessive use of antimalarials and antibiotics, due to the persistence of fever [4]. We present here the cases of two boys aged 4 and 10 years admitted for fever, which persisted after a well-conducted antimalarial and antibiotic treatment. The diagnosis of Still disease or systemic juvenile idiopathic arthritis (SJIA) was made late. Both patients were treated with a dose regressive corticosteroid therapy and methotrexate, with favourable progression.

## Patient and observation

### Case 1

A 4 years old male, admitted for fever and abdominal pains associated with a generalized tonic clonic seizure lasting approximately 5 minutes, with no ocular revulsion nor sphincteric relaxation nor postictal state. The past medical history was unremarkable and the physical examination revealed a temperature of 39.5°C. The absence of inflammatory signs in the cerebrospinal fluid (CSF) analysis and a positive thick blood smear (120 trophozoites/μl) led to the diagnosis of severe malaria (convulsion being the criterion of severity). Other blood tests such as full blood count reported 8,000 leukocytes/mm^3^ (normal) with 75% granulocytes, 164,000 platelets/mm^3^, a moderate normochromic normocytic anaemia with a haemoglobin level of 10.3 g/dl. He received parenteral antimalarial treatment (artesunate) with oral relay (artemisinin combination therapy) for 3 days supplemented with an antipyretic. After 48 hours, the patient remained febrile and three blood cultures were done. Septicaemia dose ceftriaxone was prescribed for 72 hours, yet the fever persisted. Second-line antibiotic therapy with imipenem and amikacin was given for 72 hours with persistent fever.

After 06 days, all blood cultures done were sterile. No infectious site was clearly identified and other causes of persistent fever in children, including other inflammatory diseases and haematological malignancies were investigated. Biologic workups showed: a CRP level at 48.11 mg/l, transaminases at 130.40 IU/l (normal <50) for glutamic oxaloacetic transaminase (GOT) and 40.32 IU/l (normal <35) for glutamic pyruvic transaminase (GPT), ferritinaemia at 1200.46μg/l (normal: 15-100), elevated LDH at 1576 IU/L (normal 200-500 IU/L) and elevated triglycerides. Rheumatoid and antinuclear factors, anti cyclic citrullinated peptide (CCP), blood smear and viral serologies were unremarkable. The diagnosis of Still disease was made. The patient received one-month dose regressive corticosteroid therapy, combined with methotrexate from the second week. The fever completely regressed twelve hours after the start of corticosteroid therapy, and the patient's clinical state improved considerably.

### Case 2

A 10 years old boy was admitted for a 10 days duration persistent fever and asthenia. He described an odynophagia from two days prior to the onset of fever. His past medical history was unremarkable. The physical examination revealed an altered general state, a temperature of 39°C, no sign of meningeal irritation and no clinically evident portal of entry. He had a class II splenomegaly, with micro polyadenopathies located at the inguinal region and adjoin the upper right cervical chain, with good oral hygiene. A walking lameness occurred on the 8^th^ day with painful mobilization of the right hip. He had received a well-conducted outpatient anti-malaria treatment and probabilistic antibiotherapy with amoxicillin-clavulanic acid followed by ceftriaxone at the correct dose. Biologically there was a severe inflammatory syndrome with sedimentation rate at 94mm/h, CRP at 96mg/l, leucocytosis at 13000/mm^3^ with neutrophil polynucleosis, ferritinaemia elevated to 1100 microgram/l, (normal: 15-100), the 3 sets of blood cultures were sterile and the arthrocentesis fluid was inflammatory and sterile. Viral serologies [hepatitis B, C, human immunodeficiency virus (HIV)] were negative. Triglycerides were increased at 2g/l, LDH levels at 1000IU/l (normal 200-500IU/l). Liver and kidney functions were satisfactory, the calcium phosphate levels were normal and the cytobacteriological examination of urine was unremarkable. The rheumatoid and antinuclear factors were negative.

The chest X-ray showed no infection site, the right hip ultrasound showed intra-articular effusion and the abdominal ultrasound found no lymphadenopathy. The diagnosis of Still disease was made. As soon as the first dose of corticosteroids was administered at 1mg/kg, there was a regression of the fever within 24 hours. In addition to corticosteroid therapy, bedrest was recommended during periods of relapses with a diet low in salt, sugar, fat, and the gradual addition of methotrexate at a low dose (10 mg/m^2^ of body surface area). With this treatment, the progression was favourable.

## Discussion

SJIA is part of a heterogeneous group of early arthritis in children less than 16 years of age, whose causes are unknown. Based on the clinical presentation and the biological workups, there are 07 different forms [3]. JIA can start with fever, associated with arthritis or not. Both patients presented with fever but no arthritis, although this occurred in the second patient 08 days later. Physical examination and blood tests generally help to exclude the infectious and malignant causes of fever. This is particularly important in low/middle-income countries where these infectious causes are endemic, and represent the main aetiologies of fever [5]. Faced with the persistence of fever after proper antibiotic and antimalarial treatment, it is therefore important to look for evidence of an inflammatory disease in general, and SJIA in particular.

The management of SJIA is generally multidisciplinary. The objective of this management is to guarantee, in the most severe forms, the vital and functional prognosis while limiting the inflammatory process and pain. All these using the treatment with the least risk of intolerance. A progression towards a macrophage activation syndrome is to be feared because the mortality rate linked to this complication stands at 5-8% [2]. As a result, even though non-steroidal anti-inflammatory drugs or general corticosteroids are most often the preferred treatment during the first weeks of the disease, the progression is towards the increasingly frequent and early recourse to methotrexate and even to biological treatment using anti-interleukin 1 or 6 [6].

The favorable response of patients to interleukin 1 and 6 inhibitors confirms the role of interleukin 1 in the etiopathology of SJIA. SJIA can rightly be considered an auto-inflammatory syndrome [7]. Our patients rather benefited from treatment with methotrexate. It should be noted that JIA can also occur in patients on immunosuppressive therapy [8, 9]. However, this was not the case of our patients.

## Conclusion

Cases of SJIA are rarely documented in sub-Saharan African literature. This auto inflammatory pathology with a fearsome complication is not often mentioned. Inflammatory causes should be investigated in cases of persistent fever in children in malaria endemic areas, even in the absence of osteoarticular signs. The diversity in clinical presentation shows the need for more research to be done on the pathophysiology of this condition.

## Competing interests

The authors declare no competing interests.
